# Identification and Validation of Long Non-Coding RNA LCIIAR as a Biomarker in LUAD

**DOI:** 10.3389/fonc.2022.933071

**Published:** 2022-07-04

**Authors:** Wenjun Ren, Yixiao Yuan, Xi Chen, Haoqing Zhai, Yin An, Lin Tang, Juan Wang, Dahang Zhang, Liren Zhang, Wanli Cheng, Xin Wang, Lincan Duan, Luciano Mutti, Bin Han, Ping Wang

**Affiliations:** ^1^ Department of Thoracic Surgery, The Second Affiliated Hospital of Kunming Medical University, Kunming, China; ^2^ Department of Cardiovascular Surgery, The First People’s Hospital of Yunnan Province, the Affiliated Hospital of Kunming University of Science and Technology, Kunming, China; ^3^ Department of Thoracic Surgery, The Third Affiliated Hospital of Kunming Medical University, Kunming, China; ^4^ First Department of Neurosurgery, The Second Affiliated Hospital of Kunming Medical University, Kunming, China; ^5^ First Kunming College of Life Science, University of Chinese Academy of Sciences, Beijing, China; ^6^ Department of Gastroenterology, The First People's Hospital of Yunnan Province/The Affiliated Hospital of Kunming University of Science and Technology, Kunming, China; ^7^ Sbarro Institute for Cancer Research and Molecular Medicine, Center for Biotechnology, College of Science and Technology, Temple University, Philadelphia, PA, United States; ^8^ Department of Emergency, The First People’s Hospital Yunnan Province/The Affiliated Hospital of Kunming University of Science and Technology, Kunming, China

**Keywords:** LCIIAR, lung adenocarcinoma, prognostic biomarkers, immune cell infiltration, DNA methylation

## Abstract

Lung cancer is the leading cause of cancer-related death worldwide. Therapies for lung cancer have relatively poor outcomes and need to be improved. Lung cancer immune cell infiltration associated RNA (LCIIAR) is a long noncoding RNA (lncRNA), which is overexpressed in human cancers. However, the clinical significance and functional role of LCIIAR in Lung Adenocarcinoma remain unclear. Here, we identified a novel long non-coding RNA (ENSG00000256802), termed LCIIAR (lung cancer immune cell infiltration associated lncRNA), up-regulated in lung cancer tissue and cell lines. We show that increase LCIIAR expression correlated with poor clinical stage and adverse clinical outcomes and that could also serve as an independent unfavorable prognostic factor in patients with Lung Adenocarcinima. GSEA analysis demonstrated that LCIIAR is mainly involved in the regulation of the immune response. We uncovered that elevate LCIIAR expression positively correlated with immune infiltration and immune modulator in Lung Adenocarcinoma. More importantly, we confirmed that silencing of LCIIAR expression significantly inhibits the proliferation, and migration abilities of these tumour cells. We also demonstrated that the LCIIAR/hsa-miR184/SLC16A3/CDCP1 network regulates SLC16A3/CDCP1 overexpression in and is associated with poor prognosis in this tumour. Therefore our findings revealed the critical role of LCIIAR in Lung Adenocarcinoma progression, which may also serve as a prognostic biomarker and novel therapeutic target.

## Introduction

Non-small Cell Lung Cancer (NSCLC), is the malignant tumor with hugh mortality rate and leads to a huge social and economic burden (1). As one of the main subtypes of NSCLC, Lung Adenocarcinoma (LUAD) plays a major role to cause this heavy death-toll (2). At present, the treatment methods of lung cancer mainly include surgical resection, radiotherapy and chemotherapy, targeted therapy and immunotherapy (2). However, the prognosis and five-year survival rate of patients with lung cancer are still disappointing (3). Therefore, it is very important to identify novel, sensitive and specific diagnostic markers and actionable tagets for the diagnosis and treatment of lung cancer.

As one of the main types of noncoding RNA, long-chain noncoding RNA (lncRNA) plays an important role in regulating tumor cell proliferation, cell metastasis, cell cycle, apoptosis and tumor immune escape (4). Accumulating evidence have shown that lncRNAs is involved in tumor initiation and progression by regulating oncogene related signaling pathways (5). For example, it has been show that LINC01123 is highly expressed in NSCLC and up-regulates c-Myc sponging miR-199a-5p, promoting NSCLC progression (6). Furthermore, increasing KCNQ1OT1 expression is reported to be correlated with the adverse clinical features and poor prognosis of patients with LUAD (7). Moreover, lncRNA MINCR was found to up-regulate the expression of c-Myc, leading to increasing the expression of cyclin A, and CDK2 but, on the contrary, to reduce the PARP-1, involved in lung cancer progression (8). However, the potential biological and molecular mechanisms of LCIIAR involvement in LUAD have not yet been fully elucidated.

In this study, we determine the, clinical relevance of LCIIAR in LUAD by analysis of TCGA-LUAD dataset and our clinical samples. Furthermore, we examine the relationship between LCIIAR expression and the infiltration levels of various immune cells by using ssGSEA method and GEPIA database whereas GSEA enrichment analysis has allowed determine the potential mechanism of LCIIAR in LUAD. Finally, qPCR assay has been used to detect the expression of LCIIAR in LUAD tissues and cells lines. Cell Counting Kit 8 (CCK8), colony formation, trans-well, and wound healing assays has employed to determine the biological function of LCIIAR in LUAD progression.

## Materials and Methods

### Expression and Diagnostic Value Analysis

TCGA-LUAD cohort data and corresponding clinical information of LUAD patients were downloaded from the TCGA website (https://portal.gdc.cancer.gov/repository). We determined the expression of LCIIAR in pan-cancer and the correlation between LCIIAR expression and clinical features in LUAD by TCGA database. We also examined the prognosis of LCIIAR in LUAD by using TCGA-LUAD dataset and compared the expression data (HTSeq-Counts) of the high or low lncRNA expression groups that identified differentially expressed genes (DEGs) using the DESeq2 R package (Love et al., 2014), with thresholds of |log 2-fold change (FC)| > 1.5 and adjusted p value < 0.05.

### Nomogram Construction and Evaluation

Based on the multivariate Cox analysis results, we established a nomogram to predict the prognosis of LUAD patients. According to the prognosis model, we calculated each patient’s risk score as the total score of each parameter, which could predict the prognosis of LUAD patients. The accuracy estimation of nomogram prediction was obtained from a calibration plot. It was found that the bias-corrected line in the calibration plot was close to the ideal curve (Keynesian cross), indicating a strong consistency between predicted values and observed values. The nomogram discrimination was determined using a concordance index (C-index), and 1,000 resamples were used in calculation by bootstrap approach. In this study, all statistical tests were two-tailed, with a statistical significance level of 0.05.

### GSEA Enrichment Analysis

Based on the LCIIAR expression level, we divided gene expression data into high LCIIAR and low LCIIAR groups, and each analysis included 1,000 times of gene set permutations. A function or pathway term with a false discovery rate (FDR) of less than 0.25 and a p-value of less than 0.05 was considered statistically significant (9).

### Immune Infiltration Analysis in LUAD

We used a GSVA R package to quantify immune infiltration of 24 tumor-infiltrating immune cells in tumor samples through ssGSEA. To examine the correlation between LCIIAR expression and immune cell infiltration, we conducted the analysis with GSVA R package (10). Moreover, we used the Spearman method to examine the association between LCIIAR expression and immune checkpoint-related genes

### Construction of the lncRNA-miRNA-mRNA Triple Regulatory Networks in LUAD

The Starbase online database (http://starbase.sysu.edu.cn/) was employed to examine the potential gene of miRNA-184. AnnoLnc2 (http://annolnc.gao-lab.org/) used to determine the downstream miRNAs of LCIIAR in LUAD.

### Cell Cultures

Human normal bronchial epithelial cell (BEAS-2B) and 3 human LUAD cells (H1975, A549 and H1299) were purchased from the Chinese Academy of Sciences Cell Bank (CASCB, China). All human lung cancer cell lines were cultured in RPMI-1640, with 10% fetal bovine serum.

### DNA Methylation Analysis

In order to assess the diverse DNA methylation sites in the promoter of LCIIAR in LUAD (11), we utilized MethSurv (https://biit.cs.ut.ee/methsurv/), a web tool to perform multivariable survival analysis using DNA methylation data. Furthermore we utilized the SMART (http://www.bioinfo-zs.com/smartapp/), an interactive web application for comprehensive DNA methylation analysis and visualization to examine the correlation between LCIIAR expression and DNA methylation in LUAD (12).

### Constructs, Transfection and Real-Time RT-PCR Assay

The negative control (NC) control and lncRNA LCIIAR targeting siRNA were purchased from RiboBio (China). Cells were transfected with indicated siRNA or negative control using Lipofectamine 3000, and then collected these cells for various experiments. The siRNA: GGACCTCAGACTGGGCTGATGGA. The qPCR primer used in our finding as follows: β-actin-F: AAGTTGACGTGGACATCCGC, β-actin-R: CCGGACTCGTCATACTCCTGCT, LCIIAR -F: GCTTGGGAGCAAATACATGTG, LCIIAR -R: AATCACACTATTTGAGGATGCC.

### 5-Azacytidine Treatment

Cells were plated in 10 cm culture dishes and incubated overnight, and then treated with 5 µM 5-Aza (Selleck, S1782) for one day and finally collected for the biological assays

### FISH Assay

For RNA fluorescence *in situ* hybridization (FISH) assay, Cy3-labelled LCIIAR probe was designed and synthesized by RiboBio (China), and the FISH kit (RiboBio, Fluorescent *In Situ* Hybridization Kit, Cat. C10910) was used to detect the non-coding RNA expression pattern following the manufacturer’s instructions. 4,6-diamidino-2-phenylindole (DAPI) was used to indicate nuclear. All images were obtained with an LSM880 NLO (Zeiss) confocal microscope system.

### Cell Proliferation and Cell Migration Assay

Cell proliferation and cell migration assay was performed as previously described (13). Briefly, 600 cells were seeded into a 6-well plate and cultured in a cell culture incubator. After 15 days, the cell colonies were washed 3 times using 1 x PBS, then treated with 4% paraformaldehyde fixed for 20 min and 0.1% crystal violet stained for 40 min. Briefly, to produce a wound, the monolayer cells in 6-well plate were scraped in a straight line with pipette tips. Plate was then washed with PBS to remove detached cells. Photographs of the scratch were taken at indicated time points using Nikon inverted microscope (Ti-S).

### Statistical Analysis

R software was used for all statistical analyses and plots. The correlations between clinicopathological characteristics and LCIIAR expression were evaluated using the Chi-squared test, Fisher exact test, Kruskal–Wallis (KW) test, Wilcoxon signed-rank test, Wilcoxon rank sum test, and logistic regression. Kaplan-Meier method was adopted to draw survival curves [hazard ratio (HR), 95% CI]. Through univariate and multivariate analysis combined with Cox logistic regression models, other clinical factors impacting the survival and the LCIIAR expression level were found. In **Figure 8** the analysis was conducted by GraphPad Prism 7 Software. The operating characteristic (ROC) analyzed of LCIIAR was examined by using operating characteristic (ROC) analysis the pROC package. For all figures, ns, ∗, ∗∗, ∗∗∗ indicate P >0.05, P < 0.05, P < 0.01, P < 0.001, respectively.

## Results

### LCIIAR Was Up-Regulated in LUAD

**Figure 1:**
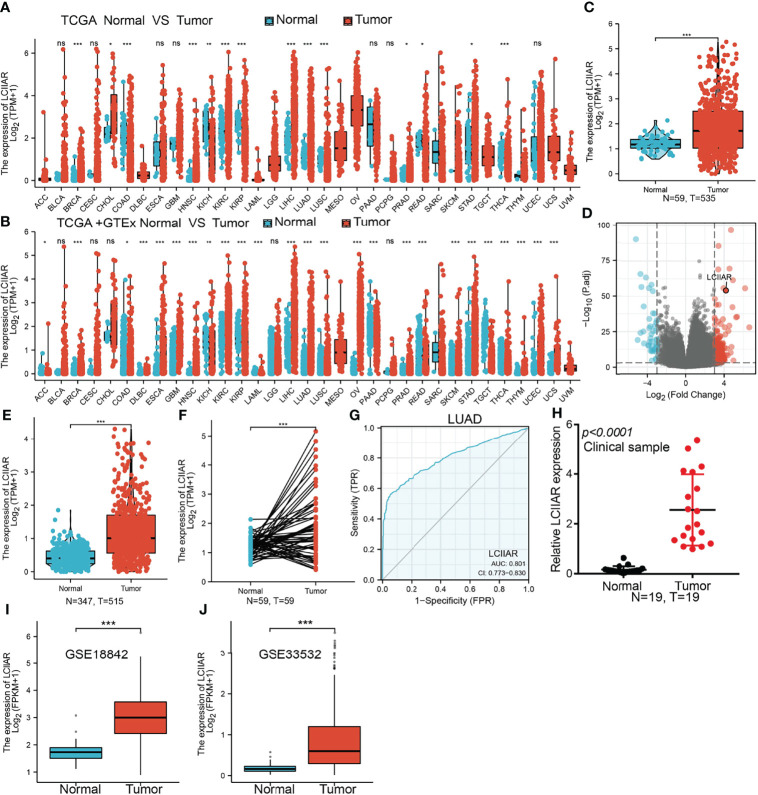
LCIIAR was highly expressed in LUAD. **(A-F)** LCIIAR expression in LUAD and LUSC was examined by TCGA/GTEX databases **(G)** ROC curve analysis of the diagnostic value of LCIIAR in LUAD **(H)** LCIIAR expression in lung cancer tissues was examined by qPCR assay. **(I, J)** LCIIAR expression in lung cancer was examined by GEO dataset, NS: p>0.05, *p < 0.05, **p < 0.01, ***p < 0.001.

To examine the LCIIAR expression in pan-cancer, we examine the expression of LCIIAR in pan-cancer by using TCGA datasets and found that lncRNA LCIIAR was differentially expression between cancer and adjacent normal tissues **(** **Figure 1A** **)** or GTEx database normal tissue **(** **Figure 1B** **)**. Additionally, we found a significantly higher LCIIAR expression in 535 lung cancer tissues than that in 59 adjacent normal tissues based on TCGA LUAD dataset **(** **Figures 1C, D** **)**. Our results also confirmed that LCIIAR was overexpressed in 59 paired LUAD tissues than in normal tissues **(** **Figures 1E, F** **).** ROC curve analysis of LCIIAR showed an AUC value of 0.801 and 0.771 in LUAD and lung squamous cell carcinoma patients, respectively **(** **Figure 1G** **)**. Finally, to confirm LCIIAR expression in lung cancer tissues, we performed qRT-PCR to detect LCIIAR in 19 paired lung cancer and adjacent normal tissues and found significantly higher LCIIAR expression in lung cancer tissues than in adjacent normal tissues **(** **Figure 1H** **).** GEO dataset also confirmed that LCIIAR was up-regulated in lung cancer compared to normal tissues **(** **Figures 1I, J** **)**.

### DNA Hypo-Methylation Induced lncRNA LCIIAR Expression in LUAD cells

**Figure 2:**
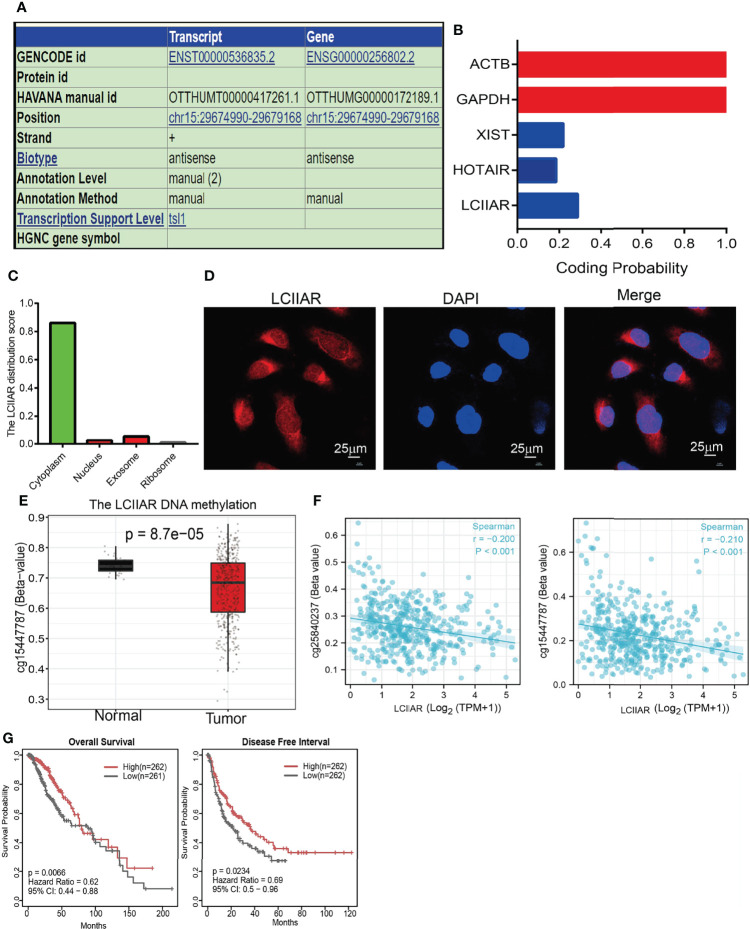
DNA methylation regulated the expression of lncRNA LCIIAR in LUAD. **(A)** The genomic attributes of LCIIAR was examined by UCSC database. **(B)** The coding potential of LCIIAR was examined by CPC2 database. **(C)** The subcellular localization of LCIIAR was predicted by LncLocater. **(D)** The sub-cellular localization of LCIIAR was examined by FISH assay in A549 cells **(E)** The methylation levels of LCIIAR in lung cancer and normal tissues. **(F)** The correlation between the methylation level and DNA expression of LCIIAR by SMART database. **(G)** Kaplan-Meier analysis of OS and DFI for the methylation level of LCIIAR in TCGA LUAD dataset.

Since there are no previous reports on the molecular characteristics of LCIIAR, we further examined the genome characteristics of lncRNA LCIIAR by using various public databases (http://genome.ucsc.edu/). The genomic attributes of LCIIAR are shown in the **Figure 2A**. LCIIAR resulted mainly located in chr15:29674990-29679168. The coding potential of LCIIAR using Coding Potential Calculator did not show coding potential **(** **Figure 2B** **)**. We also uncovered that LCIIAR was mainly localized in the cytoplasm of LUAD cells using lnclocator followed by RNA fluorescence *in situ* hybridization (FISH) **(** **Figures 2C, D** **)**. Since epigenetic dis-regulation plays an indispensable role in regulating gene expression, we also explored the mechanism by which LCIIAR was regulated, and found that the methylation of LCIIAR was significantly lower in LUAD cancerous tissues than normal tissues based on the TCGA LUAD by SMART database (http://www.bioinfo-zs.com/smartapp/) (**Figure 2E** **)** (12). Consistently, we found that the methylation levels on the specific methylation site (cg25840237 and cg15447787) within LCIIAR promoter region negatively correlated with its expression in LUAD **(** **Figure 2F** **).** In parallel, using the methSurv dataset we showed that elevated methylation levels on cg15447787 site was significantly correlated, and predicted poor survival in lung cancer patients, **(** **Figure 2G** **)** (11, 12).

### Correlation Between LCIIAR Expression and Clinic-Pathologic Characteristic

**Figure 3:**
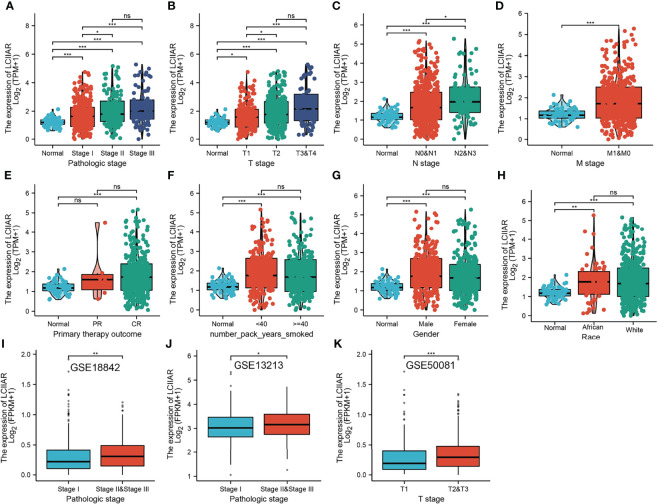
Correlation with LCIIAR expression and clinicopathologic characteristic of LUAD Correlation with LCIIAR expression and clinicopathologic characteristics, including **(A)** pathological stage, **(B–D)** TNM stage, **(E)** primary therapy outcome, **(F)** smoking, **(G)** gender and **(H)** race. **(I–K)** Correlation with LCIIAR expression and pathological stage, T stage analysis by GEO dataset. NS: p > 0.05, *p < 0.05, **p < 0.01, ***p < 0.001.

To determine the correlation between LCIIAR expression and clinic-pathological characteristics in LUAD, the samples were divided into high and low LCIIAR expression groups according to the median value. Correlation analysis was employed to identify the clinic-pathologic characteristic and LCIIAR expression level. As shown in **Table 1** and **Figure 3**, higher expression of LCIIAR was significantly correlated with smoking, gender, race and with the pathologic stage, TNM stage, and primary therapy outcome, (p<0.05). We also using GEO dataset confirmed that higher expression of LCIIAR was correlated with pathological stage and T stage **(** **Figures 3I, K** **)**.

**Table 1:** Correlation between LCIIAR expression and clinical characteristic.

Characteristic	Low expression of LCIIAR	High expression of LCIIAR	p
N	267	268	
T stage, n (%)			0.023
T1	102 (19.2%)	73 (13.7%)	
T2	137 (25.8%)	152 (28.6%)	
T3	18 (3.4%)	31 (5.8%)	
T4	8 (1.5%)	11 (2.1%)	
N stage, n (%)			0.038
N0	183 (35.3%)	165 (31.8%)	
N1	45 (8.7%)	50 (9.6%)	
N2	27 (5.2%)	47 (9.1%)	
N3	1 (0.2%)	1 (0.2%)	
M stage, n (%)			0.043
M0	175 (45.3%)	186 (48.2%)	
M1	17 (4.4%)	8 (2.1%)	
Age, median (IQR)	65 (59, 72)	67 (59, 73)	0.480

**Figure 4:**
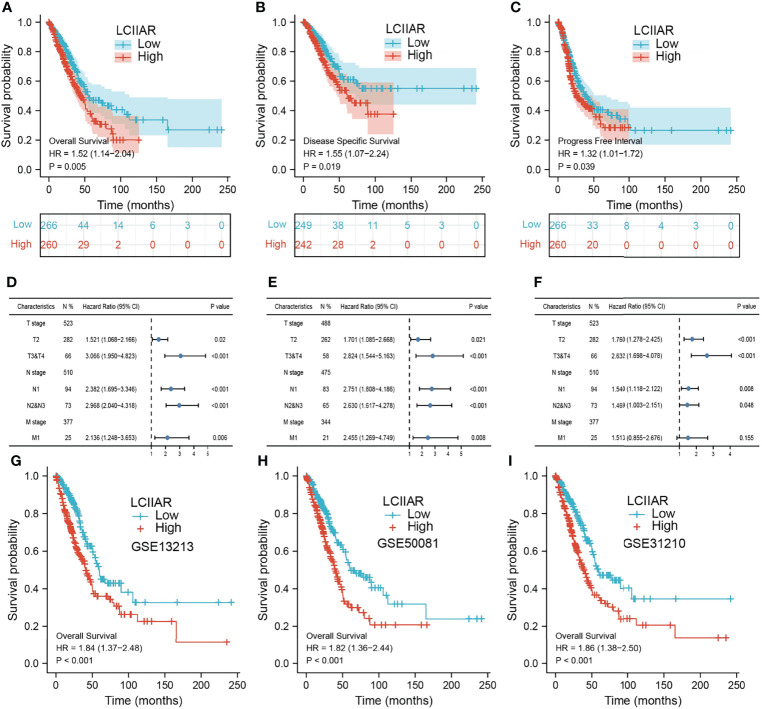
Analysis the prognosis of LCIIAR in LUAD **(A–C)** The prognosis of LCIIAR in LUAD was examined by TCGA-LUAD dataset **(D–F)** The prognosis of LCIIAR based on TNM stage subtype. **(G–I)** Validation of the overall survival of LCIIAR in lung cancer by GEO dataset.

Furthermore, Kaplan-Meier survival analysis was used to investigate the correlation between LCIIAR expression and overall survival (OS) or progression-free survival (PFS) or disease-specific survival (DSS) in the LUAD patients. Results suggested that patients with higher expression level of LCIIAR correlated with the poor overall survival, disease-specific survival, and progression-free survival **(** **Figures 4A–C** **).** Moreover, multivariable hazards models were used to evaluate the effect of expression of LCIIAR and TNM stage on overall survival, disease-specific survival, and progression free survival **(** **Figures 4D–F**). Finally, GEO dataset also show that higher expression of LCIIAR was correlated with adverse clinical outcomes in lung cancer patients **(** **Figures 4G–I** **)**.

**Figure 5:**
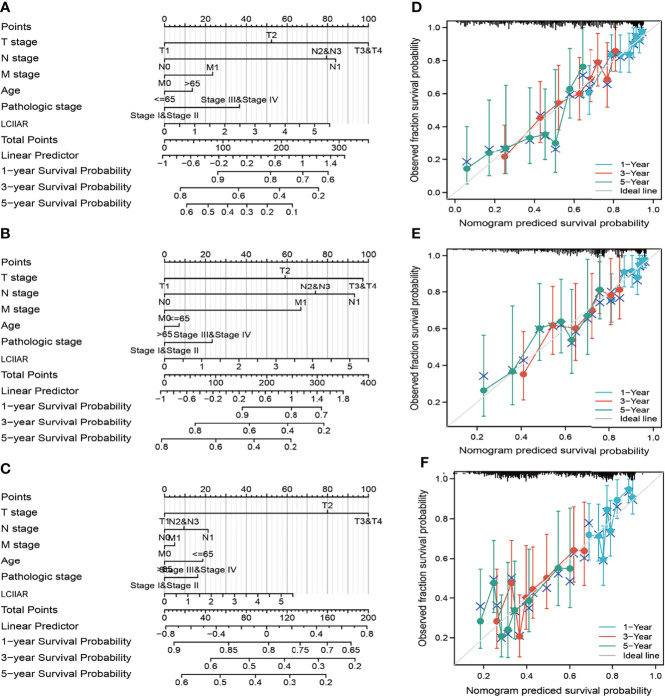
Construction and performance validation of the LCIIAR based nomogram for LUAD patients. **(A–F)** Nomogram to predict the prognosis for lung cancer patients.

As shown in the **Table 2**, patients with complete clinical data were analysed with an additional Cox regression analysis. The Cox univariate regression and multivariate analysis suggested that LCIIAR was an independent prognostic factor for overall survival in LUAD patients, along with N stage and primary therapy outcome. We also constructed the nomogram combining LCIIAR and independent clinical risk factors, and confirmed that this nomogram could be better predicted the OS, DSS, and PFS respectively **(** **Figures 5A–5F** **)**.

**Table 2:** Univariate regression and multivariate survival model of prognostic covariates in patients with lung cancer.

Characteristics	Total (N)	Univariate analysis		Multivariate analysis	
		Hazard ratio (95% CI)	P value	Hazard ratio (95% CI)	P value
T stage	523				
T1&T2	457				
T3&T4	66	2.317 (1.591-3.375)	<0.001	19.540 (3.970-96.182)	<0.001
N stage	510				
N0&N1	437				
N3&N2	73	2.321 (1.631-3.303)	<0.001	9.885 (1.269-76.984)	0.029
Pathologic stage	518				
Stage II&Stage I	411				
Stage IV&Stage III	107	2.664 (1.960-3.621)	<0.001	0.565 (0.078-4.087)	0.572
M stage	377				
M0	352				
M1	25	2.136 (1.248-3.653)	0.006	7.773 (0.752-80.311)	0.085
Primary therapy outcome	108				
SD	37				
PD	71	3.174 (1.549-6.505)	0.002	4.298 (1.170-15.788)	0.028
Race	461				
Black or African American	55				
White	406	1.443 (0.871-2.389)	0.155		
Age	516				
<=65	255				
>65	261	1.223 (0.916-1.635)	0.172		
Residual tumor	363				
R0	347				
R2&R1	16	3.879 (2.169-6.936)	<0.001	0.492 (0.072-3.361)	0.470
Gender	526				
Female	280				
Male	246	1.070 (0.803-1.426)	0.642		
Anatomic neoplasm subdivision	512				
Left	200				
Right	312	1.037 (0.770-1.397)	0.810		
Smoker	512				
No	72				
Yes	440	0.894 (0.592-1.348)	0.591		
LCIIAR	526	1.271 (1.115-1.449)	<0.001	1.712 (0.997-2.941)	0.041

### GSEA Enrichment of LCIIAR in LUAD

**Figure 6:**
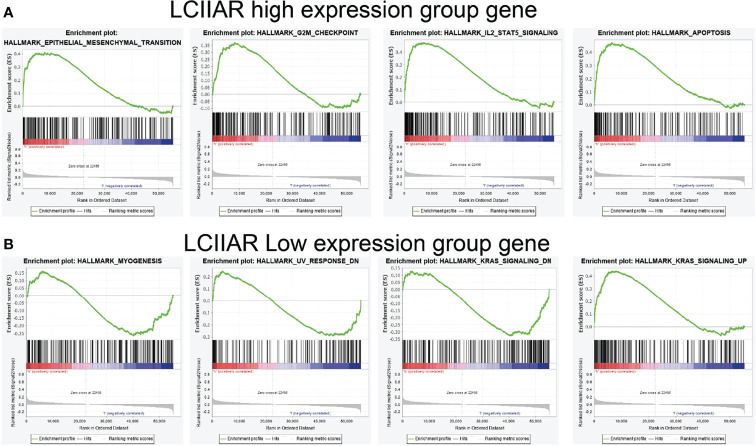
GSEA enrichment of LCIIAR **(A, B)** GSEA enrichment analysis of the potential signaling pathway of LCIIAR high and low expression group gene in LUAD.

We further determined the potential signaling pathways of LCIIAR involved in LUAD with GSEA-software analysis (9). Results indicated that in higher expression of LCIIAR group the most prominent genes mostly involved were those EMT, G2/M check point, apoptosis and IL-2 STAT5 signaling pathway **(** **Figures 6A** **).** On the contrary, the gene in the lower expression of LCIIAR group mainly involved in the Myogenesis, UV response, and KRAS signaling pathway **(** **Figures 6B** **).**

### Relationship Between LCIIAR Expression and Immune Infiltration in LUAD

**Figure 7:**
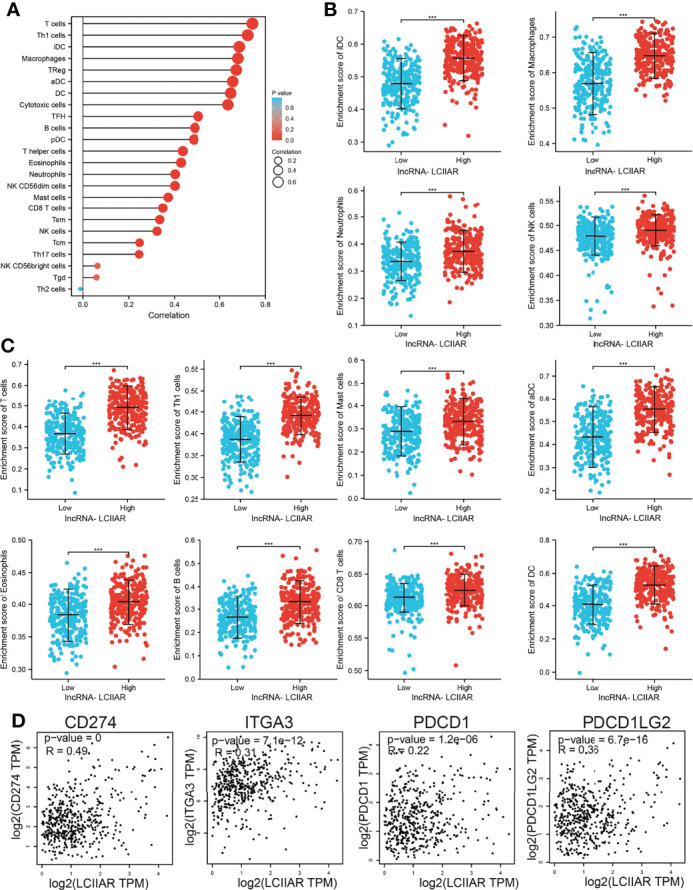
Correlation between LCIIAR expression and immune cell infiltration **(A–C)** Correlation between LCIIAR expression and various immune cell infiltration in LUAD. **(D)** Correlation between LCIIAR expression and check point-related gene in LUAD. ***p < 0.001.

We further explored the correlation between LCIIAR and immune infiltration by using Spearman’s correlation. Results demonstrated that LCIIAR positively correlates with the immune infiltration of T cells, Th1 cells, iDC, Macrophages, TReg, aDC, DC, Cytotoxic cells, TFHB cells, pDC, T helper cells, Eosinophils, Neutrophils, NK CD56dim cells, Mast cells, B cell, CD8 T cells, Tem, NK cells, Tcm, Th17 cells and NK CD56bright cells **(** **Figures 7A–C** **).** Finally, we uncovered that LCIIAR positively correlated with the expression of immune check point related genes, including CD274, PDCD1, ITGA3 and PDCD1LG2 in LUAD **(** **Figures 7D** **)**.

### Depletion of LCIIAR Inhibits LUAD Cell Growth and Migration

**Figure 8:**
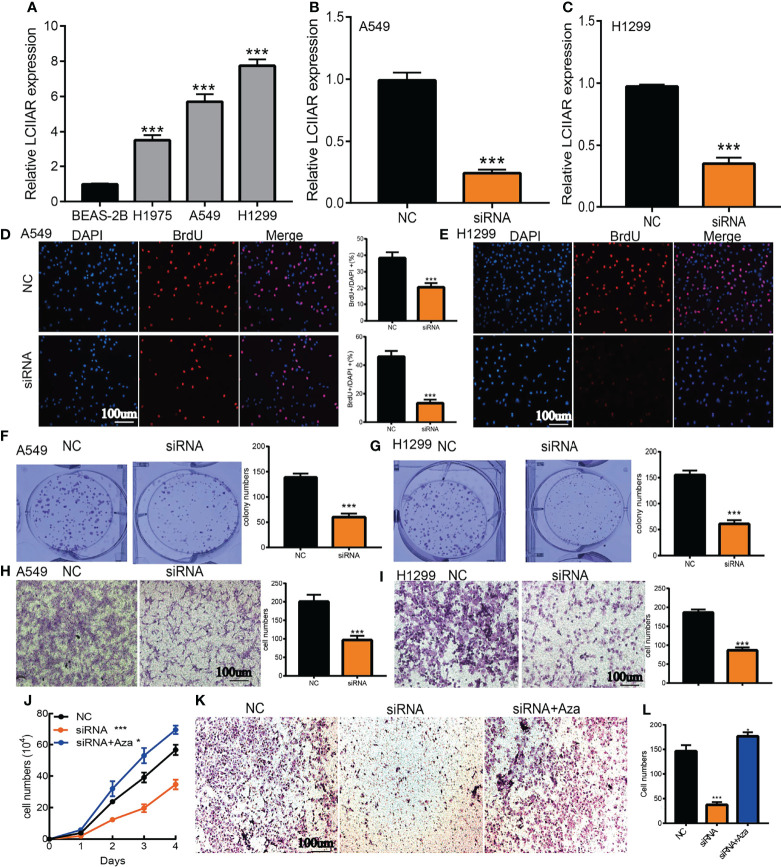
Depletion of LCIIAR inhibited the proliferation and cell migration of LUAD **(A)** The expression level of LCIIAR in LUAD cells lines and BEAS-2B. **(B, C)** Establishment of LCIIAR knockdown cell lines in A549 and H1299 verified by Real-time RT-PCR **(D–G)** Knockdown of LCIIAR significantly inhibited cell growth examined by BrdU and colony formation assays. **(H, I)** Knockdown of LCIIAR inhibited LUAD cells migration determined by transwell assays. **(J-L)** Assessment of the role of hypo-methilation in LCIIAR-mediated lung cancer proliferation and migration, scale bar=100 μm, NC=negative control, siRNA=LCIIAR siRNA *p < 0.05, ***p < 0.001.

To explore the biological function of LCIIAR, we first examined the LCIIAR expression in LUAD cell lines. We showed that LCIIAR increased in LUAD cell lines compared to BEAS-2B **(** **Figures 8A** **).** Furthermore, we used qRT-PCR assay to verify the knockdown efficiencies (**Figures 8B, C** **)**. As expected, LCIIAR knockdown inhibited LUAD cells proliferation and colony formation **(** **Figures 8D–G** **).** Next, we confirmed that knockdown of LCIIAR inhibited LUAD cell migration examined by trans-well assays **(** **Figures 8H, I** **).** To investigate whether LCIIAR-mediated lung cancer proliferation and migration are dependent on its hypo-methylation, we conducted the rescue experiment and found that 5Aza treat could overcome the cellular effect resulted from LCIIAR knockdown on the LUAD cell proliferation and migration **(** **Figures 8J–L** **)**.

### LCIIAR Related miRNAs-mRNAs Network in LUAD

**Figure 9:**
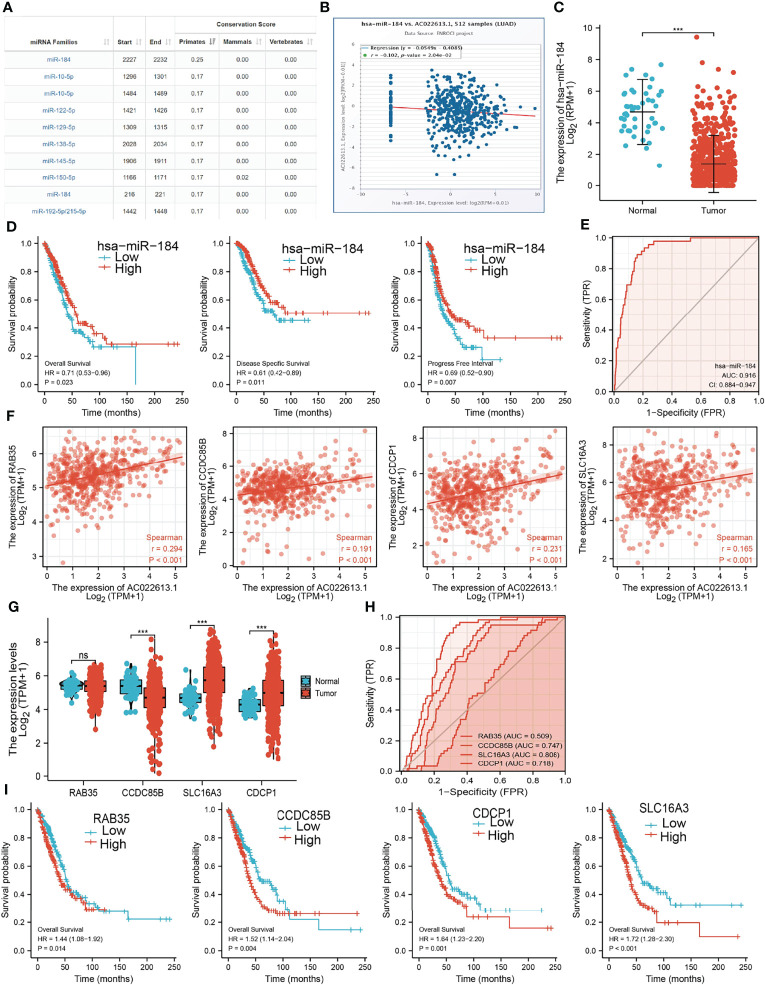
Analysis of the potential ceRNA network of of LCIIAR **(A)**The potential miRNAs of LCIIAR was determined by Anbolnc2 database. **(B)** Correlations between LCIIARexpression and miR-184 in LUAD **(C)** The expression level of miR-184 in LUAD **(D)** OS, DSS and PFS of miR-184 in LUAD. **(E)** ROC curve of miR-184 in LUAD. **(F)** Correlations between LCIIAR expression and RAB35, CCDC85B, SLC16A3, and CDCP1 in LUAD. **(G)** The RNA level of RAB35, CCDC85B, SLC16A3, and CDCP1 in LUAD. **(H)** The ROC curve of RAB35, CCDC85B, SLC16A3, and CDCP1 in LUAD. **(I)** OS of RAB35, CCDC85B, SLC16A3, and CDCP1 in LUAD, NS: p > 0.05, ***p < 0.001.

To further explore the LCIIAR-mediated downstream regulatory mechanism involved in LUAD progression, we used Annolnc2 (http://annolnc.gao-lab.org/) database that identified 10 miRNAs with potential binding with LCIIAR **(** **Figure 9A** **) (** 14). Base on the competitive endogenous RNAs theory, lncRNA should be positive correlated with mRNA and negatively correlated with miRNA. Among all the 10 miRNAs, only miRNA-184 negatively correlated with LCIIAR in LUAD **(** **Figure 9B** **)**. Moreover, we found that miRNA-184 was poorly expression in LUAD and its lower expression was correlated with poor prognosis in patients with LUAD (ROC curve AUC value: 0.916) (**Figures 9C–E** **)**. Therefore, we select miRNA-184 conducted downstream analysis.

### Identification the Potential Downstream Target of LCIIAR/miR-184 in LUAD

We further investigated the target genes of miRNA-184 that play critical roles in the progression of LUAD. Firstly, we predicted the target in StarBase, miRDB, miRWalk and miRGator (15–18). According to the prediction of target genes, we found that only 4 genes (RAB35, CCDC85B, SLC16A3, and CDCP1) were positively correlated with LCIIAR expression in LUAD **(** **Figure 9F** **)**. Furthermore, we employed the TCGA to explore the expression level and prognosis in LUAD. We found that SLC16A3 and CDCP1 were up-regulated in LUAD **(** **Figure 9G** **)**. ROC curve was utilized to examine the diagnostic value of SLC16A3, and CDCP1 in LUAD, the AUC of which were 0.718 and 0.808, respectively **(** **Figure 9H** **)**. Finally, we have found that higher expression of SLC16A3, and CDCP1 were correlated with poor prognosis in patients with LUAD **(** **Figure 9I** **)**. These results confirmed that CCDC85B, SLC16A3, and CDCP1 were a potential prognostic and diagnostic biomarker in LUAD.

## Discussion

LUAD is one of the most sinister thoracic tumors, characterized by diverse and common pathological features. Although considerable heterogeneity in LUAD has been identified by large scale genomic profiling, potential novel biomarkers that may provide new insight into the prognosis of LUAD are still in urgent demand. Our group has recently developed multiple comprehensive integrative bioinformatics methodologies to identify new biomarkers involved in hypoxic solid tumor (19, 20). Among the candidate tumor related new biomarkers the lncRNA LCIIAR, was revealed to be up-regulated in LUAD due to the hypo-methylation in its promoter region, which could be reversed by 5-Azacytidine treatment, a well-known anti-cancer drug (21). In the current work, we mainly investigated the function of LCIIAR in lung cancer. We uncovered that LCIIAR was overexpressed in LUAD cancerous tissues and LUAD cancerous cell lines. Higher expression of LCIIAR was associated with poor patient outcome. Multivariate analysis suggested that LCIIAR was an independent prognostic factor for overall survival in LUAD patients.

The subcellular localization of lncRNA directly determines the molecular mechanism and function in cancer progression thus in this study, we used online data-base analysis and conducted assay that confirmed that LCIIAR was mainly localized in the cytoplasm of LUAD cells.

It has been well confirmed that DNA methylation plays a crucial role in modulating gene expression (22). The aberrant alteration of methylation has been also

reported to be correlated with the progression of lung cancer (23). We showed that increase in DNA methylation leads to decrease in expression of LCIIAR in LUAD cells. We also uncovered that elevated methylation levels on cg15447787 site correlated with better overall survival and disease-free survival in the TCGA-LUAD cohorts. In prognosis analysis, increased LCIIAR was associated with adverse overall survival, disease-specific survival, and disease-specific survival. The Multivariate analysis showed that high expression of LCIIAR is associated with a more aggressive, metastatic stage of lung cancer, and with poor patient outcome.

It has been found that lncRNA JPX promoted the metastasis of lung cancer *via* sponging the miR-33a-5p, up-regulated Twist1 expression, led to activating Wnt/β-catenin signaling (13). Song et al. found that SNHG1 was increases in cervical cancer tissues and depletion of SNHG1 inhibits cell proliferation and migration as well as invasiveness in cervical cancer cells (24). In this study, we showed that LCIIAR mainly participated in the signaling pathway, including the EMT, G2/M check point, apoptosis and IL-2 STAT5 signaling pathway.

The tumor microenvironment (TME) plays a significant role in cancer progression and immune escape (25). However, to date, there have been no studies on the function of LCIIAR in the TME. For tumor immune cell infiltration, we found that in LUAD LCIIAR positively correlates with the immune infiltration of T cells, Th1 cells, iDC, Macrophages, TReg, aDC, DC, Cytotoxic cells, TFHB cells, pDC, T helper cells, Eosinophils, Neutrophils, NK CD56dim cells, Mast cells, B cell, CD8 T cells, Tem, NK cells, Tcm, Th17 cells and NK CD56bright cells. Immune-checkpoint inhibitors targeting PD-1 or PD-L1 have already substantially improved the outcomes of patients with many types of cancer, but only 20-40% of patients benefit from these therapy (26). Our results confirmed that LCIIAR expression was significantly positively correlated with the expression of CD274, LAG3, PDCD1, and PDCD1LG2, in LUAD. Currently, there are still no studies examine whether LCIIAR is correlated with cancer progression. We found that LCIIAR was up-regulated in LUAD cells and knock down of LCIIAR expression significantly inhibited cell proliferation and migration of LUAD.

The down-stream mechanisms of lncRNAs have also been widely investigated in cancer (27). CeRNAs are a common molecular regulatory mechanism of lncRNAs, and they have been intensively reported in different cancer types (28). According to the ceRNA hypothesis, lncRNAs can form a sponge with miRNAs to regulate the expression of target genes at the mRNA level (29). Recent studies have confirmed that ceRNAs have significant roles in cancer pathogenesis by altering the expression of key tumorigenic or tumor suppressive genes (29).

The main finding of this study was the identification of a prognosis-related ceRNA regulatory network (LCIIAR/hsa-miR-184/SLC16A3/CDCP1) in LUAD. In the ceRNA regulatory network, hsa-miR-184 was significantly negatively correlated with LCIIAR expression, while LCIIAR was significantly positively correlated with SLC16A3/CDCP1 expression. In addition, SLC16A3 and CDCP1 were significantly overexpressed in LUAD tissues compared to normal tissues, and survival analysis revealed that the high expression group had a poorer prognosis compared to the low expression group. On the contrary hsa-miR-184 exhibited low expression in LUAD tissues compared to normal tissue and survival analysis revealed that the low expression group had a poorer prognosis compared to the high expression group. These results consistently suggest that LCIIAR/hsa-miR-184/SLC16A3/CDCP1 is a poor prognosis-associated ceRNA regulatory network in NSCLC.

## Conclusions

Our study confirmed that LCIIAR promotes the lung cancer progression and may serve as a potential biomarker for the diagnosis of LUAD.

## Data Availability Statement

Publicly available datasets were analyzed in this study. This data can be found here: TCGA website (https://portal.gdc.cancer.gov/repository).

## Ethics Statement

The human studies were reviewed and approved by The Second Affiliated Hospital of Kunming Medical University, Kunming, China. Written informed consent from the [patients/ participants OR patients/participants legal guardian/next of kin] was not required to participate in this study in accordance with the national legislation and the institutional requirements.

## Author Contributions

WR, YY, XC, HZ, and YA designed this work and performed related assay, LT, JW, DZ, LZ, WC, XW, and LD analyzed the data. BH, PW and LM supervised and wrote the manuscript. All authors have read and approved the final version of the manuscript.

## Funding

This study was sponsored by the Applied Basic Research Project of Yunnan provincial Science and Technology Department and Kunming Medical University (grant No. 2020001AY070001-117 and 202001AY070001-130). The Open Project of The First People's Hospital of Yunnan Province Clinical Medicine Center (2021LCZXXF‐XZ03).

## Conflict of Interest

The authors declare that the research was conducted in the absence of any commercial or financial relationships that could be construed as a potential conflict of interest.

## Publisher’s Note

All claims expressed in this article are solely those of the authors and do not necessarily represent those of their affiliated organizations, or those of the publisher, the editors and the reviewers. Any product that may be evaluated in this article, or claim that may be made by its manufacturer, is not guaranteed or endorsed by the publisher.
